# Neuroticism, physical activity, and cognitive functioning in a population-based cohort of older adults

**DOI:** 10.1186/s12877-023-04399-8

**Published:** 2023-11-06

**Authors:** Pankaja Desai, Todd Beck, Kristin R. Krueger, Robert S. Wilson, Denis A. Evans, Kumar B. Rajan

**Affiliations:** 1 Rush Institute for Healthy Aging, Rush University Medical Center, Triangle Office Building, 1700 W Van Buren, Suite 245, Chicago, IL 60612 USA; 2 Rush Alzheimer’s Disease Center, Rush University Medical Center, Chicago, IL USA; 3 Department of Neurology, University of California at Davis, Davis, CA USA

**Keywords:** Neuroticism, Physical Activity, Cognitive Function, Cognitive Decline

## Abstract

**Background:**

Little is known about how physical activity influences the relationship between neuroticism and cognitive function and cognitive decline.

**Methods:**

Data from the Chicago Health and Aging Project (CHAP) was utilized to conduct this study. CHAP is a population-based cohort study of chronic conditions in older adults. Participants completed in-home interviews cycles of three years from 1993–2012. Mixed effects regression models were conducted to test the associations between physical activity, neuroticism, and the interaction between neuroticism and physical activity on outcomes: global cognitive function, global cognitive decline, episodic memory, decline in episodic memory, perceptual speed, and decline in perceptual speed. Stratified mixed effects regression models by physical activity level were conducted to test the associations between neuroticism and global cognitive function and global cognitive decline.

**Results:**

A total of 7,685 participants were eligible for this study. Participants were 62% female and 64% African American. We found statistically significant associations for the interaction of high physical activity and neuroticism on baseline global cognitive function (β = 0.017 (SE = 0.007), *p* = .010) and on the interaction of neuroticism and high physical activity on baseline episodic memory (β = 0.020 (SE = .009), *p* = .021) and on decline in episodic memory over time (β = -0.003 (SE = .001),* p* = .039).

**Conclusion:**

Higher physical activity lessened the association between higher neuroticism and poor cognitive outcomes.

## Introduction

Neuroticism is an established personality trait that is an important target for public health interventions [1]. It is strongly correlated with and predicts several physical and mental health conditions and comorbidities, as well as health and mental health care utilization [2]. Neuroticism is defined by characteristics such as anxiety, depression, anger, variability of emotion, susceptibility to irritation, and greater self-consciousness. Individuals with more neuroticism tend to have difficulty managing stress and often experience feelings of being threatened, overwhelmed, and hopeless during day-to-day life occurrences [1].

Less neuroticism is associated with increased physical activity participation and decreased sedentary behavior and inactivity [3]. Individuals with an inactive or less active lifestyle are more likely to have decreased conscientiousness, openness, agreeableness, and extraversion [4]. Greater neuroticism is also associated with cognitive decline and poor cognitive performance [5]. Limited information exists regarding the relationship between physical activity, neuroticism and cognitive decline. Engaging in regular physical activity across different life stages helps to expand cognitive or brain reserve, priming the brain for resiliency, despite cognitive difficulties or pathology in the brain [6]. Persons with decreased neuroticism seem to have enhanced pathological resilience or cognitive functioning given their neuropathology [7]. We did not find papers which examined whether or not physical activity moderates or mediates the association between neuroticism and cognitive decline. In this study, we evaluate the personality trait of neuroticism because of its heterogeneity in the population, because it is strongly correlated with many physical and mental health conditions, compared to other personality traits, and because of its potential for public health impact [2]. We test the interaction of baseline physical activity and baseline neuroticism on baseline cognitive function and cognitive decline over time. We hypothesize that high baseline physical activity level weakens the association between baseline neuroticism and baseline cognitive function, compared to little physical activity level at baseline. We also hypothesize that high baseline physical activity slows rate of decline in the association between baseline neuroticism and cognitive decline over time, compared to little physical activity level at baseline.

## Methods

We analyzed data from the Chicago Health and Aging Project (CHAP), which is a population-based cohort study that examines health conditions in community-dwelling African American and White older adults. Recruitment of study participants occurred using door-to-door census. In-home interviews were completed in three-year cycles from 1993 to 2012. CHAP has a total of 10,802 participants. Clinical evaluations were completed with a stratified, random sample of approximately 33% of total CHAP participants. CHAP study design is further described elsewhere [8]. A total of 7,685 participants were eligible for this study and completed at least two time points of cognitive performance measurement. CHAP is approved by the Rush University Medical Center IRB, and CHAP participants provided written informed consent.

### Neuroticism

Neuroticism was measured using items from the NEO Five-Factor Inventory. Participants were asked to rate each of the following items on a scale of 1 (*Strongly disagree)* to 5 (*Strongly agree*): *I often feel tense and jittery; I am not a worrier; I often feel helpless and want someone else to solve my problems; I often get angry at the way people treat me.*Ratings were summed to calculate a total score between 4–20 and recoded 0–16. The second item was reverse coded [9]. Baseline measurement of neuroticism was used for this study. For the figure only, neuroticism was categorized as low at approximately the 10^th^ percentile and high at approximately the 90^th^ percentile.

### Physical activity

Physical activity participation was measured using items from the 1985 US Health Interview Survey. Participants reported their participation in the following activities *in the past 14 days*: *walking for exercise, jogging or running, gardening or yard work, dancing, calisthenics or general exercise, golf, bowling, bicycle riding, swimming or water exercises,* and *other exercises, sports or physically active hobbies* [10, 11]. The number of *times* the activity was performed was multiplied by the mean number of *minutes* for *each occasion* the activity was performed *in the past 14 days* [10, 11]. This total was divided by two to determine the number of minutes per week or seven days per activity. The minutes per activity were then summed across all items to obtain the total number of minutes per week of physical activity participation. Physical activity was categorized into little activity, medium activity, and high activity. Participants with little activity responded to at least four items and had 0 min per week of participation. Participants with medium activity engaged in a total of less than or equal to150 minutes per week, and participants with high activity engaged in a total which was greater than 150 min per week. Physical activity was categorized because of the large number of participants with little activity. The cut off of 150 min per week also parallels the American College of Sports Medicine recommendation of a minimum of 150 min per week of activity at moderate levels for adults [12]. Baseline measurement of physical activity was used for this study.

### Demographic characteristics and chronic medical conditions

Demographic characteristics include age, sex, race, and education level and were obtained using items from the 1990 US Census Bureau [13]. Chronic medical conditions consisted of summing if participants reported diabetes, stroke, hypertension, cancer, fractured or broken hip, and cardiovascular conditions [14, 15]. Depressive symptoms were measured using a summary score of the modified version of the Center for Epidemiologic Studies-Depression (CESD), which consisted of ten items [16–18].

### Cognitive function

Cognitive functioning was assessed utilizing the East Boston Memory Test: Immediate Recall and Delayed Recall, which measures episodic memory, the Symbol Digit Modalities Test (modified, oral version), which measures perceptual speed, and the Mini-Mental State Examination (MMSE) [19–22]. The z-score for each measure was determined using the mean and standard deviation of the baseline score. Global cognitive function score was calculated by taking the mean of z-scores from all measures [23].

#### Statistical analysis

Descriptive analysis was conducted in total and by physical activity category. Mixed effects regression models were conducted to test the associations between neuroticism as a continuous variable and physical activity as a categorical variable on the longitudinal trajectory of each of three outcomes: global cognitive function, episodic memory, and perceptual speed. For each outcome, two models were fit to examine the effects neuroticism and physical activity levels separately. A third model then included both effects into one model, and finally, a fourth model added a neuroticism by physical activity interaction term. Mixed effects regression models were also conducted, stratified by the three levels of physical activity, which examined the associations between neuroticism and global cognition. All models adjusted for age, race, sex, education, medical conditions, depressive symptoms, time, and interactions of each with time. Models have person-specific intercepts and slopes and an unstructured correlation matrix. Level of statistical significance used for this analysis was *p* < 0.05. The variance inflation factor was calculated for all predictor variables in the models. Analysis was done in SAS version 9.4. A figure was developed using R version 4.2.1. to plot global cognitive decline over time among participants with high neuroticism, little physical activity, high neuroticism, high physical activity, low neuroticism, little physical activity, and low neuroticism, high physical activity.

## Results

### Descriptive analysis

Table 1 describes baseline characteristics of the study sample in total and by physical activity category. The total study sample had a mean age of 72 years, included 62% female participants and 64% African American participants, and had a mean of 12 years of education. At baseline, the mean neuroticism score was 5.35 out of 16, and the mean minutes of physical activity per week was 75. Participants with little physical activity had a mean neuroticism score of 5.77 compared to a mean of 5.01 for participants with high physical activity.

**Table 1:** Baseline sample characteristics in total and by physical activity category

Variable	Overall *N* = 7,685^1^	0 Minutes *N* = 2285^1^	> 0 Minutes to < = 150 Minutes *N* = 2585^1^	> 150 Minutes *N* = 2815^1^	*p*-value^2^
Age	72.2 (6.2)	72.6 (6.7)	72.4 (6.3)	71.7 (5.6)	< 0.001
Education	12.4 (3.5)	11.6 (3.4)	12.4 (3.5)	13.1 (3.6)	< 0.001
Medical Conditions	1.11 (0.98)	1.23 (1.03)	1.11 (0.98)	1.01 (0.93)	< 0.001
CESD-10	1.46 (1.93)	1.98 (2.21)	1.41 (1.88)	1.08 (1.59)	< 0.001
Global cognition	0.30 (0.70)	0.15 (0.78)	0.30 (0.67)	0.41 (0.62)	< 0.001
Episodic Memory	0.29 (0.83)	0.17 (0.91)	0.29 (0.80)	0.39 (0.76)	< 0.001
Perceptual Speed	0.35 (0.93)	0.17 (0.93)	0.36 (0.92)	0.50 (0.91)	< 0.001
Neuroticism	5.35 (2.27)	5.77 (2.38)	5.34 (2.26)	5.01 (2.12)	< 0.001
Physical Activity (minutes)	75 (0, 240)	0 (0, 0)	60 (30, 105)	345 (225, 540)	< 0.001
Study Time (years)	6.9 (3.5, 9.6)	6.5 (3.4, 9.3)	7.1 (3.5, 9.8)	7.0 (3.6, 10.1)	< 0.001
Female	4,803 (62%)	1,638 (72%)	1,670 (65%)	1,495 (53%)	< 0.001
African American	4,929 (64%)	1,723 (75%)	1,670 (65%)	1,536 (55%)	< 0.001

*CESD-10*   Modified Center for Epidemiologic Studies-Depression Scale

^1^Mean (SD); Median (IQR); n (%)

^2^One-way ANOVA; Kruskal–Wallis Rank Sum test; Pearson’s Chi-squared test

### Mixed effects regression models: global cognition, episodic memory, perceptual speed

Table 2 includes the results of four models of longitudinal global cognitive function examining associations between 1. neuroticism, 2. physical activity, 3. neuroticism and physical activity, and 4. the interaction of neuroticism and physical activity. Betas represent standard deviation units. Betas that do not include time represent units of cognitive function at baseline. Terms that include time are interpreted as units of annual rate of change in cognitive function. Model 1 shows statistically significant associations between neuroticism and baseline global cognitive function (β = -0.036 (SE = 0.003), *p* < 0.000), *p* = 0.002). Model 2 has statistically significant associations between high physical activity and global cognitive decline (β = 0.006 (SE = 0.003), *p* = 0.018). Model 3 indicates statistically significant associations between neuroticism and baseline level (β = -0.035 (SE = 0.003), *p* < 0.000) and between high physical activity decline in global cognition (β = 0.006 (SE = 0.003), *p* = 0.028). Model 4 shows that the interaction of high physical activity and neuroticism (β = 0.017 (SE = 0.007), *p* = 0.010) on baseline global cognitive function was statistically significant.

**Table 2:** Associations between physical activity, neuroticism, and global cognitive function and decline

	Model 1			Model 2			Model 3			Model 4		
Participants	7613			7626			7608			7608		
Total observations	25,338			25,372			25,327			25,327		
	β	SE	*p*-value	β	SE	*p*-value	β	SE	*p*-value	β	SE	*p*-value
Intercept	0.493	0.014	0.000	0.464	0.018	0.000	0.478	0.018	0.000	0.484	0.018	0.000
Time	-0.049	0.002	0.000	-0.054	0.003	0.000	-0.053	0.003	0.000	-0.053	0.003	0.000
Age	-0.034	0.001	0.000	-0.034	0.001	0.000	-0.034	0.001	0.000	-0.034	0.001	0.000
Age*time	-0.004	0.000	0.000	-0.004	0.000	0.000	-0.004	0.000	0.000	-0.004	0.000	0.000
Sex	-0.124	0.013	0.000	-0.108	0.013	0.000	-0.126	0.013	0.000	-0.126	0.013	0.000
Sex*time	0.006	0.002	0.004	0.006	0.002	0.004	0.005	0.002	0.010	0.005	0.002	0.010
Race	-0.358	0.014	0.000	-0.354	0.015	0.000	-0.355	0.014	0.000	-0.355	0.014	0.000
Race*time	-0.001	0.002	0.540	-0.001	0.002	0.649	-0.001	0.002	0.730	-0.001	0.002	0.724
Education	0.062	0.002	0.000	0.066	0.002	0.000	0.062	0.002	0.000	0.062	0.002	0.000
Education*time	-0.001	0.000	0.123	0.000	0.000	0.184	-0.001	0.000	0.095	-0.001	0.000	0.083
Medical conditions	0.009	0.006	0.165	0.004	0.006	0.525	0.009	0.006	0.164	0.009	0.006	0.155
Medical conditions*time	-0.002	0.001	0.042	-0.002	0.001	0.042	-0.002	0.001	0.058	-0.002	0.001	0.055
CESD	-0.010	0.003	0.000	-0.018	0.003	0.000	-0.010	0.003	0.000	-0.010	0.003	0.000
CESD*time	-0.002	0.000	0.000	-0.002	0.000	0.000	-0.002	0.000	0.000	-0.002	0.000	0.000
Neuroticism	-0.036	0.003	0.000				-0.035	0.003	0.000	-0.046	0.005	0.000
Neuroticism*time	-0.001	0.001	0.211				-0.001	0.001	0.259	0.000	0.001	0.642
Medium PA				0.030	0.016	0.054	0.023	0.016	0.134	0.015	0.016	0.343
High PA				0.029	0.016	0.069	0.017	0.016	0.280	0.010	0.016	0.547
Medium PA*time				0.003	0.003	0.208	0.003	0.003	0.236	0.004	0.003	0.159
High PA*time				0.006	0.003	0.018	0.006	0.003	0.028	0.006	0.003	0.017
Medium PA*neuroticism										0.013	0.007	0.060
High PA*neuroticism										0.017	0.007	0.010
Medium PA*neuroticism*time										-0.001	0.001	0.319
High PA*neuroticism*time										-0.002	0.001	0.189

Time is reported in years

Models adjusted for age, sex, race, education, medical conditions, CESD, and each of their interactions with time

*Medium PA* Medium Physical Activity, *High PA* High Physical Activity, *CESD* Modified Center for Epidemiologic Studies-Depression Scale

* refers to interaction

Similar analysis for the four models were conducted with episodic memory as an outcome in Table 3 and with perceptual speed as an outcome in Table 4. In Table 3, models 1 and 3 indicate that the association between neuroticism and baseline episodic memory is statistically significant (β = -0.042 (SE = 0.004), *p* < 0.000). Model 4 shows a statistically significant association between the interaction of neuroticism and high physical activity on baseline episodic memory (β = 0.020 (SE = 0.009), *p* = 0.021) and on decline in episodic memory over time (β = -0.003 (SE = 0.001), *p* = 0.039). In Table 4, models 1 and 3 indicate statistically significant associations between neuroticism and perceptual speed at baseline (β = -0.033 (SE = 0.004), *p* < 0.000). Variance inflation factor values for predictors in the models were between 1.000–2.000, indicating low correlations among predictors.

**Table 3:** Associations between physical activity, neuroticism, and episodic memory and decline in episodic memory

	Model 1			Model 2			Model 3			Model 4		
Participants	7585			7598			7580			7580		
Total observations	25,104			25,138			25,093			25,093		
	β	SE	*p*-value	β	SE	*p*-value	β	SE	*p*-value	β	SE	*p*-value
Intercept	0.389	0.018	0.000	0.369	0.023	0.000	0.383	0.023	0.000	0.390	0.023	0.000
Time	-0.032	0.003	0.000	-0.037	0.004	0.000	-0.036	0.004	0.000	-0.037	0.004	0.000
Age	-0.033	0.001	0.000	-0.033	0.001	0.000	-0.033	0.001	0.000	-0.033	0.001	0.000
Age*time	-0.003	0.000	0.000	-0.003	0.000	0.000	-0.003	0.000	0.000	-0.003	0.000	0.000
Sex	-0.092	0.017	0.000	-0.072	0.017	0.000	-0.094	0.017	0.000	-0.094	0.017	0.000
Sex*time	0.007	0.003	0.005	0.007	0.003	0.008	0.007	0.003	0.009	0.007	0.003	0.009
Race	-0.282	0.019	0.000	-0.277	0.019	0.000	-0.279	0.019	0.000	-0.278	0.019	0.000
Race*time	-0.004	0.003	0.184	-0.004	0.003	0.226	-0.003	0.003	0.255	-0.003	0.003	0.249
Education	0.049	0.003	0.000	0.054	0.003	0.000	0.049	0.003	0.000	0.049	0.003	0.000
Education*time	0.000	0.000	0.688	0.000	0.000	0.697	0.000	0.000	0.612	0.000	0.000	0.547
Medical conditions	0.033	0.008	0.000	0.028	0.008	0.001	0.033	0.008	0.000	0.033	0.008	0.000
Medical conditions*time	-0.002	0.001	0.197	-0.002	0.001	0.223	-0.002	0.001	0.245	-0.002	0.001	0.233
CESD	-0.005	0.004	0.224	-0.015	0.004	0.000	-0.004	0.004	0.236	-0.004	0.004	0.301
CESD*time	-0.003	0.001	0.000	-0.002	0.001	0.000	-0.003	0.001	0.000	-0.003	0.001	0.000
Neuroticism	-0.042	0.004	0.000				-0.042	0.004	0.000	-0.053	0.006	0.000
Neuroticism*time	0.000	0.001	0.619				0.000	0.001	0.556	0.002	0.001	0.043
Medium PA				0.014	0.021	0.504	0.006	0.020	0.768	-0.003	0.021	0.898
High PA				0.024	0.021	0.253	0.011	0.021	0.598	0.003	0.021	0.899
Medium PA*time				0.005	0.003	0.149	0.005	0.003	0.152	0.006	0.003	0.072
High PA*time				0.006	0.003	0.064	0.006	0.003	0.073	0.007	0.003	0.033
Medium PA*neuroticism										0.013	0.009	0.144
High PA*neuroticism										0.020	0.009	0.021
Medium PA*neuroticism*time										-0.002	0.001	0.125
High PA*neuroticism*time										-0.003	0.001	0.039

Time is reported in years

Models adjusted for age, sex, race, education, medical conditions, CESD, and each of their interactions with time

*Medium PA* Medium Physical Activity, *High PA* High Physical Activity, *CESD* Modified Center for Epidemiologic Studies-Depression Scale

* refers to interaction

**Table 4:** Associations between physical activity, neuroticism, and perceptual speed and decline in perceptual speed

	Model 1			Model 2			Model 3			Model 4		
Participants	7308			7315			7304			7304		
Total observations	24,205			24,225			24,196			24,196		
	β	SE	p-value	β	SE	*p*-value	β	SE	*p*-value	β	SE	*p*-value
Intercept	0.772	0.017	0.000	0.765	0.022	0.000	0.778	0.022	0.000	0.779	0.022	0.000
Time	-0.059	0.002	0.000	-0.061	0.003	0.000	-0.060	0.003	0.000	-0.060	0.003	0.000
Age	-0.045	0.001	0.000	-0.045	0.001	0.000	-0.045	0.001	0.000	-0.045	0.001	0.000
Age*time	-0.002	0.000	0.000	-0.002	0.000	0.000	-0.002	0.000	0.000	-0.002	0.000	0.000
Sex	-0.181	0.016	0.000	-0.163	0.016	0.000	-0.179	0.016	0.000	-0.179	0.016	0.000
Sex*time	0.005	0.002	0.012	0.005	0.002	0.014	0.005	0.002	0.024	0.005	0.002	0.023
Race	-0.656	0.018	0.000	-0.657	0.018	0.000	-0.658	0.018	0.000	-0.658	0.018	0.000
Race*time	0.013	0.002	0.000	0.013	0.002	0.000	0.013	0.002	0.000	0.014	0.002	0.000
Education	0.090	0.002	0.000	0.093	0.002	0.000	0.090	0.002	0.000	0.090	0.002	0.000
Education*time	-0.002	0.000	0.000	-0.002	0.000	0.000	-0.002	0.000	0.000	-0.002	0.000	0.000
Medical conditions	-0.023	0.008	0.004	-0.028	0.008	0.001	-0.023	0.008	0.004	-0.023	0.008	0.004
Medical conditions*time	-0.004	0.001	0.000	-0.004	0.001	0.000	-0.004	0.001	0.000	-0.004	0.001	0.000
CESD	-0.019	0.003	0.000	-0.025	0.003	0.000	-0.019	0.003	0.000	-0.019	0.003	0.000
CESD*time	-0.001	0.001	0.006	-0.001	0.001	0.064	-0.001	0.001	0.008	-0.001	0.001	0.008
Neuroticism	-0.033	0.004	0.000				-0.033	0.004	0.000	-0.035	0.006	0.000
Neuroticism*time	0.000	0.001	0.401				0.000	0.001	0.439	-0.001	0.001	0.567
Medium PA				0.011	0.020	0.565	0.005	0.020	0.817	0.002	0.020	0.904
High PA				-0.004	0.020	0.829	-0.016	0.020	0.425	-0.018	0.020	0.383
Medium PA*time				0.000	0.003	0.918	-0.001	0.003	0.855	-0.001	0.003	0.864
High PA*time				0.004	0.003	0.147	0.004	0.003	0.181	0.004	0.003	0.196
Medium PA*neuroticism										0.004	0.008	0.650
High PA*neuroticism										0.004	0.008	0.671
Medium PA*neuroticism*time										0.000	0.001	0.832
High PA*neuroticism*time										0.001	0.001	0.618

Time is reported in years

Models adjusted for age, sex, race, education, medical conditions, CESD, and each of their interactions with time

*Medium PA* Medium Physical Activity, *High PA* High Physical Activity, *CESD* Modified Center for Epidemiologic Studies-Depression Scale

* refers to interaction

### Mixed effects regression models: stratified analysis by physical activity level

Table 5 shows associations between neuroticism and global cognition and global cognitive decline, stratified by physical activity level: little, medium, and high. There are inverse associations between neuroticism and baseline cognitive function that are statistically significant within each physical activity level: little (β = -0.044 (SE = 0.006), *p* < 0.000), medium (β = -0.032 (SE = 0.005), *p* < 0.000), and high (β = -0.032 (SE = 0.005), *p* < 0.000).

**Table 5:** Stratified analysis by physical activity level: associations between neuroticism and global cognitive function and decline

	Little PA			Medium PA			High PA		
Participants	2253			2556			2799		
Total observations	7094			8636			9597		
	β	SE	*p*-value	β	SE	*p*-value	β	SE	*p*-value
Intercept	0.457	0.032	0.000	0.526	0.023	0.000	0.504	0.020	0.000
Time	-0.059	0.005	0.000	-0.047	0.004	0.000	-0.047	0.003	0.000
Age	-0.036	0.002	0.000	-0.035	0.002	0.000	-0.028	0.002	0.000
Age*time	-0.004	0.000	0.000	-0.003	0.000	0.000	-0.004	0.000	0.000
Sex	-0.090	0.029	0.002	-0.140	0.022	0.000	-0.141	0.018	0.000
Sex*time	0.003	0.005	0.539	0.010	0.004	0.005	0.003	0.003	0.333
Race	-0.338	0.033	0.000	-0.369	0.024	0.000	-0.351	0.020	0.000
Race*time	0.009	0.005	0.086	-0.005	0.004	0.214	-0.002	0.003	0.484
Education	0.074	0.004	0.000	0.061	0.003	0.000	0.056	0.003	0.000
Education*time	0.000	0.001	0.629	-0.001	0.001	0.033	0.000	0.001	0.721
Medical conditions	0.002	0.012	0.878	-0.006	0.011	0.603	0.032	0.010	0.001
Medical conditions*time	0.000	0.002	0.838	-0.002	0.002	0.221	-0.003	0.002	0.061
CESD	-0.005	0.005	0.264	-0.012	0.005	0.008	-0.012	0.005	0.007
CESD*time	-0.004	0.001	0.000	-0.002	0.001	0.005	-0.001	0.001	0.069
Neuroticism	-0.044	0.006	0.000	-0.032	0.005	0.000	-0.032	0.005	0.000
Neuroticism*time	0.001	0.001	0.436	-0.001	0.001	0.289	-0.001	0.001	0.102

Time is reported in years

Models adjusted for age, sex, race, education, medical conditions, CESD, and each of their interactions with time

*Medium PA* Medium Physical Activity, *High PA* High Physical Activity, *CESD* Modified Center for Epidemiologic Studies-Depression Scale

* refers to interaction

### Global cognitive decline over time

**Fig. 1:**
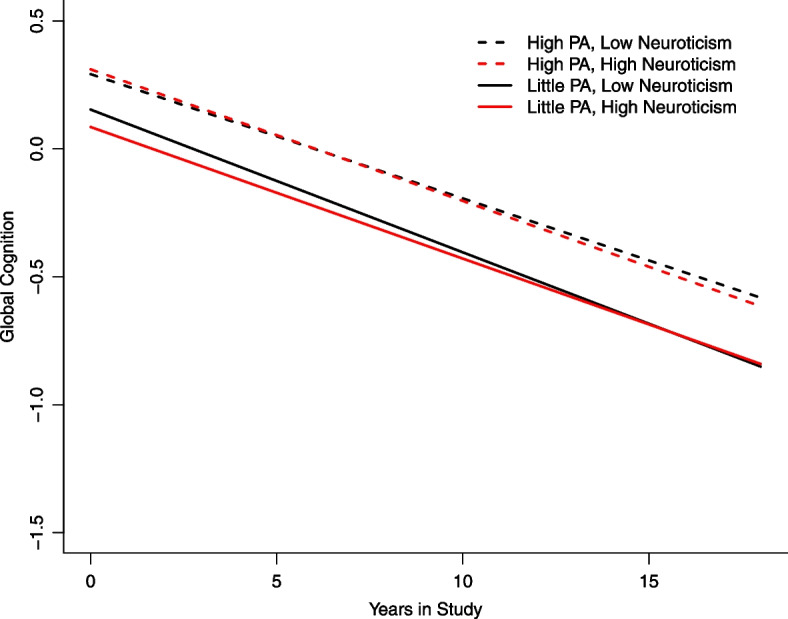
Global cognitive decline by level of neuroticism and physical activity. This plot characterizes global cognitive decline over time in years as determined by mixed effects regression models. Each line in the plot represents one of four groups of participants: a) high physical activity and low neuroticism = black, dashed line; b) high physical activity and high neuroticism = red, dashed line; c) little physical activity and low neuroticism = black, solid line; and d) high physical activity and high neuroticism = red, dashed line

Figure 1 describes global cognitive decline over the number of years in the study. The figure shows that individuals who have high or low levels of neuroticism, who engage in high physical activity level begin at a similar level of cognitive function which is higher than for individuals who engage in little physical activity. Participants with high or low levels of neuroticism have a rate of global cognitive decline that is similar and is a slower rate compared to individuals who engage in little physical activity.

## Discussion

The study results show that high physical activity differentially influences the association between neuroticism and global cognitive function and episodic memory at baseline and decline in episodic memory over time. The study findings indicate that the association between neuroticism and global cognitive function is dependent on physical activity level. A high level of physical activity attenuates the association between neuroticism and global cognitive decline.

Little work has been done to examine how physical activity, which is a modifiable risk factor, influences the relationship between neuroticism and cognitive decline in older adults. However, studies have been conducted which evaluate the role of physical activity in the associations between characteristics of neuroticism, such as depression or anxiety, and cognitive decline. Data from the National Health and Nutrition Examination Survey (2011–2014) was used to assess how physical activity impacts the relationship between depression and cognitive function in older adults. Results showed that participating in 150 min per week of physical activity at moderate to vigorous intensity helps to modify the association between depression and cognitive function and that physical activity may protect cognitive function from depression [24]. The English Longitudinal Study of Ageing (ELSA) found that decreased physical activity served as a mediator in the relationship between depression and cognitive decline [25]. Another study cross-sectionally examined depression as a moderator in the association between neuroticism and cognitive function in an older adult clinical population and found that depression did not moderate this association. However, authors indicated that receiving a diagnosis of depression may increase likelihood of poor cognitive functioning among individuals with increased neuroticism and that more research is needed to understand these relationships [26]. We build upon existing work by examining moderation of physical activity in the association between neuroticism and cognitive decline over multiple follow up time points and over several years.

More research has focused on evaluating associations between depressive symptoms and Alzheimer’s disease compared to symptoms of anxiety. Yet, anxiety is approximately 40% prevalent in individuals with Alzheimer’s disease. Symptoms of anxiety can also occur in early stages of experiencing cognitive impairment and can exacerbate symptoms, especially if management strategies are not in place [27]. Data from the Mayo Clinic Study of Aging was utilized to evaluate the longitudinal relationships between physical inactivity and neuropsychiatric symptoms and incidence of mild cognitive impairment. Findings indicated an interaction that was additive of physical inactivity, disruption of sleep, clinically diagnosed depression or clinically diagnosed anxiety on MCI incidence [28]. Another study focused on participants with recent Parkinson’s Disease diagnosis who were part of the Parkinson's Progression Marker Initiative (PPMI). The study results showed that having anxiety in the early part of the disease trajectory may lead to decreased engagement in physical activity and in turn, contribute to cognitive decline [29].

However, the relationship between neuroticism and physical activity is complex. Healthy neuroticism is described as neuroticism interacting with conscientiousness. In specific situations, neuroticism can benefit health. Analysis of data from the studies in the Integrative Analysis of Longitudinal Studies of Aging and Dementia (IALSA) network found healthy neuroticism to be predictive of increased physical activity participation and that conscientiousness may weaken the relationship between neuroticism and physical activity [30].

There are several limitations to this study. Physical activity was self-reported and therefore, susceptible to recall and social desirability biases [31]. Intensity of physical activity was not measured and several items in the physical activity measure pertain to formal exercise, which may potentially explain why almost 30% of participants reported little activity. We also did not measure healthy neuroticism. A subset of total CHAP participants were selected for this study. Strengths of this study include its longitudinal, population-based study design and it sample size with approximately 64% African American participants represented.

## Conclusions and future directions

In conclusion, engaging in physical activity may weaken the relationship between high neuroticism and poor cognitive functioning. Future research should examine associations by type of physical activity, focus on specific items in the NEO measure, and evaluate the roles of neuroticism and conscientiousness on physical activity behavior. We also plan to test for race, sex, and education differences in the associations over time. Understanding demographic differences is important for physical activity program planning. Additional research is needed to determine the frequency, intensity, and types of physical activity, as well as health behavior change strategies needed to reduce the impact of neuroticism on cognitive decline.

## Data Availability

The datasets used and/or analysed during the current study available from the corresponding author on reasonable request. Please contact the corresponding author to make a request.
